# Disruption of gender‐affirming health care, and COVID‐19 illness, testing, and vaccination among trans Australians during the pandemic: a cross‐sectional survey

**DOI:** 10.5694/mja2.52169

**Published:** 2023-11-23

**Authors:** Sav Zwickl, Tomi Ruggles, Alex FQ Wong, Ariel Ginger, Lachlan M Angus, Kalen Eshin, Teddy Cook, Ada S Cheung

**Affiliations:** ^1^ The University of Melbourne Melbourne VIC; ^2^ Austin Health Melbourne VIC; ^3^ La Trobe University Melbourne VIC; ^4^ ACON Sydney NSW

**Keywords:** COVID‐19, Gender identity, Healthcare disparities, Vaccination

## Abstract

**Objectives:**

To assess rates of disruption of gender‐affirming health care, of coronavirus disease 2019 (COVID‐19) illness, testing, and vaccination, and of discrimination in health care among Australian trans people during the COVID‐19 pandemic.

**Design, setting:**

Online cross‐sectional survey (1–31 May 2022); respondents were participants recruited by snowball sampling for TRANSform, an Australian longitudinal survey‐based trans health study, 1 May – 30 June 2020.

**Participants:**

People aged 16 years or older, currently living in Australia, and with a gender different to their sex recorded at birth.

**Main outcome measures:**

Proportions of respondents who reported disruptions to gender‐affirming health care, COVID‐19 illness, testing, and vaccination, and positive and negative experiences during health care.

**Results:**

Of 875 people invited, 516 provided valid survey responses (59%). Their median age was 33 years (interquartile range, 26–45 years); 193 identified as women or trans women (37%), 185 as men or trans men (36%), and 138 as non‐binary (27%). Of 448 respondents receiving gender‐affirming hormone therapy, 230 (49%) reported disruptions to treatment during the pandemic; booked gender‐affirming surgery had been cancelled or postponed for 37 of 85 respondents (44%). Trans‐related discrimination during health care was reported by a larger proportion of participants than in a pre‐pandemic survey (56% *v* 26%). COVID‐19 was reported by 132 respondents (26%), of whom 49 reported health consequences three months or more after the acute illness (37%; estimated Australian rate: 5–10%). Three or more COVID‐19 vaccine doses were reported by 448 participants (87%; Australian adult rate: 70%).

**Conclusions:**

High rates of COVID‐19 vaccination among the trans people we surveyed may reflect the effectiveness of LGBTIQA+ community‐controlled organisation vaccination programs and targeted health promotion. Training health care professionals in inclusive services for trans people could improve access to appropriate health care and reduce discrimination.

**The known**: Poorer health among trans people and barriers to health care were recognised in Australia prior to the coronavirus disease 2019 (COVID‐19) pandemic.

**The new**: COVID‐19 vaccination rates were markedly higher among respondents to our May 2022 survey of trans people than for Australia overall (three doses: 87% *v* 70%), but the proportion who reported persistent symptoms three months or more after COVID‐19 was larger (37% *v* 5–10%). Disruptions to gender‐affirming health care during the pandemic were frequent, as was discrimination during health care (eg, misgendering).

**The implications**: A targeted public health response is needed to reduce discrimination in health care and to improve inclusive care for trans people.

Transgender and gender‐diverse (trans) people — those whose gender is different to their sex recorded at birth — include trans men, trans women, non‐binary people, and people of other cultural gender identities, including Sistergirls and Brotherboys. We have previously described the poorer health status and barriers to care for trans Australians prior to the coronavirus disease 2019 (COVID‐19) pandemic.^1^, ^2^ Systemic discrimination and socio‐economic disadvantage contribute to substantial mental distress; in a 2017–18 Australian survey, 73% of trans people reported a history of depression, and 43% had attempted suicide.^2^, ^3^ In the United States, the risk of death by suicide is more than twice as high for trans people as for people whose gender identity matches their sex recorded at birth,^4^ and differences in all‐cause mortality have also been reported.^5^

Social restrictions early in the COVID‐19 pandemic had a negative impact on the mental health of trans Australians.^6^ In contrast to many countries, the first eighteen months of the pandemic were characterised by relatively low numbers of COVID‐19 cases and deaths because of widespread social restrictions.^7^ Our community survey during the first three months of the pandemic (1019 respondents) identified major disruptions to health care for trans people; gender‐affirming surgery was cancelled or postponed for 61% of those for whom it was planned, and this experience was associated with a 56% increase in the likelihood of thoughts of self‐harm or suicide.^8^

Little is known about the health care experiences of Australian trans people later in the pandemic. Overseas, social stigma, fear of discrimination, and mistrust of health care professionals contributed to COVID‐19 vaccine hesitancy among trans people.^9^ Further, trans people often report chronic illness and mood disorders,^4^, ^5^, ^10^ both known risk factors for post‐COVID‐19 syndromes (long COVID).^11^, ^12^ Disruptions to gender‐affirming health care would also be anticipated, as well as increased discrimination in health care, the result of difficulty in providing training to health care providers in trans‐affirming health care, compounded by COVID‐19‐related priority shifts. We therefore assessed rates of disruption of gender‐affirming health care, of COVID‐19 illness, testing, and vaccination, and of discrimination in health care during the pandemic in a cross‐sectional survey of Australian trans people.

## Methods

We recruited participants for our online cross‐sectional survey from among the participants in TRANSform, an ongoing longitudinal Australian trans health survey‐based study, launched in May 2020. TRANSform participants — people aged 16 years or more, currently living in Australia, and with a gender different to their sex as recorded at birth — were recruited by non‐probability snowball sampling; invitations to participate were posted on social media and shared by Australian trans community support groups and organisations. The research protocol was retrospectively published on the University of Melbourne research website (https://doi.org/10.26188/24002469.v1; 22 August 2023).

TRANSform participants complete an enrolment survey that collects basic demographic information and an email address, and are then invited by email to participate in two or three sub‐studies each year. The respondents to the survey reported in this article had been recruited for TRANSform and completed the first online survey on the impact of COVID‐19 on the trans community during 1 May – 30 June 2020. The survey, developed in March 2020 in response to clinical reports of poor mental health and suicide among trans people during the pandemic, was not piloted or validated; the survey and its results have been described in detail elsewhere.^6^, ^8^

Our second COVID‐19 survey assessed the more recent impact of the pandemic on trans people. Of the 1019 participants who completed the initial online survey, 875 were invited in emails including an individualised link to complete the follow‐up survey online during 1–31 May 2022 (144 people could not be contacted or had ended participation in TRANSform). The survey preamble stated that completing the survey implied consent to participation and the publication of survey results.

The survey was designed collaboratively by our core team of researchers, who are members of the Australian trans community, with support from clinicians specialised in health care for trans people. Survey data were collected and managed using REDCap electronic data capture tools hosted at the University of Melbourne.

The survey comprised multiple choice and optional free text questions about the general demographic characteristics of the respondents, disruptions to gender‐affirming hormone therapy and surgery, telehealth experiences, discrimination in health care, and COVID‐19 testing, infection, and vaccination during the pandemic (Supporting Information). We report respondent characteristics as counts and proportions (categorical variables) or medians with interquartile ranges (IQRs) (continuous variables).

### Ethics approval

The study was approved by the Austin Health Human Research Ethics Committee (HREC/57155/Austin‐2019), the ACON Research Ethics Review Committee (2020/03), and the Thorne Harbour Health Community Research Endorsement Panel (THH/CREP 20‐006).

## Results

Of the 875 participants invited to complete the survey, 516 provided valid responses (59%). Their median age was 33 years (IQR, 26–45 years); 193 identified as women or trans women (37%), 185 as men or trans men (36%), and 138 as non‐binary (27%) (Box 1).

Box 1 **Demographic characteristics of the 516 respondents to the second TRANSform online survey on the impact of the COVID‐19 pandemic on trans people in Australia (May 2022)**

**Table:** 

Characteristic	Respondents
Age group (years)	
18–25	125 (24%)
26–35	171 (33%)
36–45	95 (18%)
46–55	55 (11%)
56–65	48 (9%)
66–75	19 (4%)
76–82	3 (1%)
Employment status*	
Full‐time employment	211 (41%)
Part‐time employment	88 (17%)
Casual employment	77 (15%)
Unemployed	79 (15%)
Pension	51 (10%)
House duties	22 (4%)
Volunteer	20 (4%)
Retired	22 (4%)
Student	87 (17%)
Sex registered at birth	
Male	221 (42.8%)
Female	288 (55.8%)
Unsure	1 (< 1%)
Prefer not to say	6 (1%)
Variation in sex characteristics (intersex)	
No	450 (87.2%)
Yes	10 (2%)
Unknown	56 (11%)
Gender identity	
Woman/trans woman	193 (37.4%)
Man/trans man	185 (35.9%)
Non‐binary	138 (26.7%)
Indigenous status	
Aboriginal	19 (4%)
Torres Strait Islander	0
Non‐Indigenous	490 (95%)
Prefer not to say	7 (1%)
State/territory of residence	
Australian Capital Territory	26 (5%)
New South Wales	130 (25%)
Northern Territory	6 (1%)
Queensland	63 (12%)
South Australia	29 (6%)
Tasmania	12 (2%)
Victoria	201 (39%)
Western Australia	49 (10%)

COVID‐19 = coronavirus disease 2019.

* More than one response possible.

### Disruptions to gender‐affirming health care

Of the 448 participants currently receiving gender‐affirming hormone therapy (87%), 230 (49%) reported at least one type of disruption to treatment during the pandemic, most frequently difficulties with regard to access (eg, disruption of pharmacy supply; 153 respondents, 34%) or obtaining prescriptions (67 respondents, 15%). Of the 85 participants who had booked gender‐affirming surgery since 1 May 2020 (16%), surgery had been cancelled or postponed for 37 (44%); disruptions were most frequently reported by respondents in Victoria (24 respondents) and NSW (six respondents), the states most affected by COVID‐19‐related restrictions. Disruptions to post‐surgery care were reported by 40 participants who had undergone gender‐affirming surgery since mid‐2020 (42%) (Box 2).

Box 2 **The May 2022 TRANSform online survey on the impact of the COVID‐19 pandemic on trans people in Australia: disruptions to gender‐affirming health care and telehealth during the COVID‐19 pandemic**

**Table:** 

Characteristic	Respondents
People currently receiving gender‐affirming hormone therapy*	448
Difficulty with access (eg, disrupted pharmacy supply)	153 (34%)
Difficulty obtaining prescriptions	67 (15%)
Difficulty administering hormones (eg, finding somewhere for providing injection)	60 (13%)
Process for commencing hormone therapy delayed	18 (4%)
People with gender‐affirming surgery booked since 1 May 2020	85
Surgery postponed or cancelled	37 (44%)
Surgery proceeded as planned	48 (56%)
People receiving post‐gender‐affirming surgery care since 1 May 2020	96
Disruptions experienced	40 (42%)
No disruptions experienced	53 (55%)
Unsure	12 (3%)
Used telehealth services	
Yes	471 (91%)
No	43 (8%)
Unsure	3 (1%)
People who reported telehealth experiences*	470
Telehealth more accessible than in‐person appointments	372 (79%)
Telehealth just as effective or more effective than in‐person appointments	145 (31%)
Telehealth less accessible than in‐person appointments	60 (13%)
Telehealth less effective than in‐person appointments	205 (43.6%)
Unsure	12 (3%)

* Multiple responses possible.

### Telehealth

A total of 471 participants had used telehealth services during the pandemic (91%), compared with 30.8% of Australians during 2021–22.^13^ Of those who had used telehealth services, 372 (79%) reported that telehealth appointments were more accessible than in‐person consultations, 205 (44%) that they were less effective than in‐person consultations, and 145 (31%) that they were just as or more effective (Box 2). In free text survey responses, participants reported that telehealth services improved access to care for many neurodivergent trans people, trans people with disabilities, and those living in regional and remote areas. However, privacy was a problem, particularly for people living in unsupportive households.

### Discrimination in health care

A total of 287 participants (56%) reported at least one incident of trans‐related discrimination in health care, most frequently misgendering (eg, incorrect pronouns; 209 respondents, 41%) and questions related to being trans when seeking care for an unrelated medical problem (133 respondents, 26%). Non‐binary participants more frequently reported most types of discrimination than trans men and trans women (Box 3).

Box 3 **The May 2022 TRANSform online survey on the impact of the COVID‐19 pandemic on trans people in Australia: discrimination in health care, by gender**

**Table:** 

Characteristic	Trans men	Trans women	Non‐binary	Total
Total number of people	185	193	138	516
Misgendering (eg, incorrect pronouns)	49 (27%)	70 (36%)	90 (65%)	209 (40.5%)
Asked inappropriate questions about being trans when seeking medical care	49 (27%)	43 (22%)	41 (30%)	133 (25.8%)
Deadnaming (eg, using legal rather than chosen name)	28 (15%)	43 (22%)	51 (37%)	122 (23.6%)
Delayed health care (eg, not taking health complaints seriously)	24 (13%)	23 (12%)	31 (23%)	78 (15%)
Inappropriate comments about being trans	23 (12%)	24 (12%)	24 (17%)	71 (14%)
Told gender‐affirming health care not a priority during the pandemic	18 (10%)	12 (6%)	10 (7%)	40 (8%)
Denial of health care (eg, refusing care because of trans status)	10 (5%)	11 (6%)	10 (7%)	31 (6%)
Laughed or joked about	8 (4%)	11 (6%)	8 (6%)	27 (5%)

### COVID‐19 testing, infections, and vaccination

Of 512 respondents to the question, 417 (81%) had been tested for COVID‐19 at least once in a testing facility, hospital, or other clinic, of whom 307 (74%) reported one or more trans‐affirming experiences and 81 (19%) one or more negative experiences during testing (Box 4).

Box 4 **The May 2022 TRANSform online survey on the impact of the COVID‐19 pandemic on trans people in Australia: COVID‐19 testing (512 responses, 99%)**

**Table:** 

Characteristic	Respondents
COVID‐19 testing at a testing facility, hospital, or clinic	
Yes	417 (81.4%)
No	93 (18.6%)
Unsure	1 (< 1%)
Prefer not to say	1 (< 1%)
Positive experiences at COVID‐19 testing*	
Gender respected	239 [57.3%]
Expression respected	209 [50.1%]
Name and pronouns respected	251 [60.2%]
Negative experiences at COVID‐19 testing*	
Gender not respected	[11%]
Expression not respected	27 [7%]
Name and pronouns not respected	52 [13%]
Test result loss/delay believed related to name/gender marker mismatch	18 [4%]
Denial of testing believed to be related to trans status	1 [< 1%]

COVID‐19 = coronavirus disease 2019.

* Multiple responses possible.

Three or more COVID‐19 vaccine doses were reported by 448 of 513 respondents (87%; all Australians: 70%^14^); seven (1%) had received no vaccine doses (all Australians: fewer than 5%^14^). Of the 505 people who had received at least one vaccine dose, 416 (82%) reported at least one type of trans‐affirming experience and 84 (17%) at least one negative experience. Difficulty proving vaccination status because of name or gender mismatches on legal documents was reported by 29 participants (6%) (Box 5).

Box 5 **The May 2022 TRANSform online survey on the impact of the COVID‐19 pandemic on trans people in Australia: COVID‐19 vaccination (513 responses, 99%)**

**Table:** 

Characteristic	Respondents
COVID‐19 vaccination doses	
None	7 (1%)
At least one	505 (98.4%)
At least two	503 (98.0%)
At least three	448 (87.3%)
Prefer not to say	1 (< 1%)
Positive experiences at COVID‐19 vaccination*	
Gender respected	326 [64.6%]
Expression respected	285 [56.4%]
Name and pronouns respected	311 [61.6%]
Vaccination certification correct	327 [64.8%]
Negative experiences at COVID‐19 vaccination*	
Gender not respected	43 [9%]
Expression not respected	26 [5%]
Name and pronouns not respected	62 [12%]
Vaccination certification had incorrect name/gender marker	29 [6%]
Need to follow up on vaccination certification because of delays attributed to name/gender marker mismatch	5 [1%]
Difficulty proving vaccination status because of name/gender mismatch on legal documents	
Yes	29 [6%]
No	456 [91.2%]
Unsure	11 [2%]
Prefer not to say	4 [< 1%]
No response	5 [1%]

COVID‐19 = coronavirus disease 2019.

* Multiple responses possible.

One or more COVID‐19 illnesses (with or without test confirmation) were reported by 132 participants (26%); 46% of Australian blood donors were seropositive for COVID‐19 in June 2022.^15^ Long term health consequences of COVID‐19 (eg, fatigue, brain fog three or more months after acute illness) were reported by 49 of 132 participants (37%) (Box 6); an estimated 5–10% of Australians who have had COVID‐19 report such symptoms.^11^

Box 6 **The May 2022 TRANSform online survey on the impact of the COVID‐19 pandemic on trans people in Australia: COVID‐19 illness (510 responses, 99%)**

**Table:** 

Characteristic	Respondents
COVID‐19 illness	
Positive COVID‐19 test result	119 (23.3%)
Symptoms consistent with COVID‐19 but not tested	13 (2.5%)
No	353 (69.2%)
Unsure	24 (4.7%)
Prefer not to say	1 (< 1%)
Long term health consequences of COVID‐19 (three months or more)	
Yes	49 [37%]
No	39 [30%]
Unsure	43 [33%]
Prefer not to say	1 [1%]

COVID‐19 = coronavirus disease 2019.

## Discussion

In our May 2022 cross‐sectional community survey of trans people, the proportion who had received three or more COVID‐19 vaccine doses (87%) was larger than for all Australians (70%). Further, the proportion of respondents who reported having had COVID‐19 (26%) was smaller than the proportion of seropositive blood donors in Australia (46%). However, long term symptoms of COVID‐19 (three or more months after infection) were reported by 37% of respondents who had had COVID‐19, considerably higher than the 5–10% estimate for all Australians.^11^ Discrimination in health care was frequently reported, including misgendering, reported by 41% of respondents. More than 40% of those who used gender‐affirming hormone therapy or had booked surgery reported pandemic‐related disruptions. Telehealth was more accessible than in‐person appointments for 79% of respondents.

Respondents to our earlier (May/June 2020) survey often reported cancellation or postponement of appointments and gender‐affirming surgery, as well as closed patient waiting lists for general, specialist, and allied health services.^8^ The responses to our second survey confirm that trans people also experienced disruptions and barriers to gender‐affirming health care in Australia later in the pandemic. Disruptions to gender‐affirming hormone therapy were reported by 49% of respondents using it, particularly reduced access to hormones (eg, disrupted pharmacy supply), reported by 34%. COVID‐19‐related cancellation or postponement of gender‐affirming surgery was reported by 44% of respondents seeking it in 2022, still a major problem but down from the 61% who reported disruptions during the first three months of the pandemic.^8^ The proportion who reported disruptions to care after gender‐affirming surgery (42%) was also lower than in 2020 (62%).^8^ These findings illustrate the continuing impact of disruptions of elective surgery in 2022, including restrictions of surgical procedures and staff shortages, which affected all elective surgery, not just gender‐affirming surgery. For people who sought gender‐affirming surgery overseas, travel restrictions and its increased cost would have been additional barriers. The impact of these disruptions is likely to continue into the foreseeable future.

As timely access to gender‐affirming health care reduces the incidence of depression and suicidal thoughts and improves quality of life for those who seek such care,^16^, ^17^, ^18^, ^19^ COVID‐19‐related disruptions have implications for the mental health of trans people. Our earlier survey, for example, found that the likelihood of thoughts of self‐harm or suicide was 56% greater for people who experienced cancelled or postponed gender‐affirming surgery.^8^ Access to timely and affordable gender‐affirming health care should accordingly be improved.

Expansion of telehealth services during the pandemic led to a significant increase in their use.^20^ Telehealth services had been used by 91% of survey respondents during the pandemic, and they were described as more accessible than in‐person consultations by 79% of those who used them. Neurodivergent trans people, trans people with disabilities, and those living in regional or remote areas benefited from improved access to care via telehealth services. However, only 31% of telehealth users described their consultations as just as or more effective than in‐person consultations, compared with 62% of Australians who described telehealth consultations as just as good as or better than in‐person consultations.^21^ The efficacy of telehealth may be hampered by the inability to perform physical examinations, technological difficulties, and less effective communication than in face‐to‐face consultations.^21^ Privacy was also raised as a concern when using telehealth, particularly by respondents living in unsupportive households.

In our pre‐pandemic survey of trans people, 26% reported discrimination in health care.^2^ A larger proportion of respondents to our May 2022 survey reported discrimination; 56% listed one or more incidents of trans‐related discrimination while seeking health care during the pandemic. The increase may reflect the continued difficulty in providing training in trans‐affirming health care, compounded by priorities shifting to COVID‐19‐related professional development. Misgendering was the most frequently reported form of discrimination (41%); the use of correct names, pronouns, and inclusive language can be powerfully affirming and reduce hesitancy to seek medical care.^22^ Training in trans‐affirming health care should be improved in continuing professional development and university curricula for primary, allied health, and specialised care.

Overseas research has suggested that the risk of COVID‐19 is higher for lesbian, gay, bisexual, transgender, queer (or questioning), intersex, asexual, and other gender and sexual minority (LGBTIQA+) people because of their employment in large numbers in areas such as hospitality and retail.^23^, ^24^ However, the proportion of respondents to our survey who reported having had COVID‐19 (26% in May 2022) was smaller than the seropositivity rate among Australian blood donors (46% in June 2022).^14^ General COVID‐19 social restrictions and mask requirements probably reduced infection of people in exposed occupations. Higher vaccination rates, unemployment, and social isolation among people in the trans community may also have depressed infection rates.

Long term health consequences were reported by 37% of respondents who had had COVID‐19, a proportion markedly higher than that for all Australians (5–10%).^11^ The larger proportion may be related to the high prevalence among trans people of chronic illness and mood disorders,^10^ risk factors for severe COVID‐19 and long COVID.^11^, ^12^

Concern has been raised overseas about vaccine hesitancy among trans people. However, 87% of our respondents reported receiving three or more COVID‐19 vaccine doses, a larger proportion than for all Australians (31 May 2022: 70%).^17^ The evidently lower level of hesitancy may reflect greater vaccine accessibility in Australia than in some other countries, and the success of targeted vaccination campaigns and promotions, such as the Victorian Government *#FabJab* initiative with its pop‐up LGBTIQA+ vaccination clinics^25^ and specific advice on the TransHub website in New South Wales.^26^

### Limitations

Non‐probability snowball recruitment for the TRANSform surveys and the response rate for the current survey (overall: 59%) limit the representativeness of our respondents and consequently the generalisability of our findings. Online recruitment may explain the large proportion of younger people among our respondents, whose experiences may not reflect those of older trans people or trans people who did not participate in the survey, including people who are less computer proficient, live in regional and remote areas with limited internet access, or have difficulty with English.

The predominance of respondents from Victoria and New South Wales also characterised earlier Australian trans community surveys.^2^ Recall bias may have influenced our findings. Further, our survey respondents may have been particularly engaged with health care, perhaps explaining their high vaccination rate. National population data for many variables were too limited for comparison with our findings.

Longer term consequences of COVID‐19 were self‐reported, limiting comparisons with long COVID in the broader community. However, long COVID is not well defined, and the broad nature of the survey question was consistent with contemporary knowledge of the syndrome and the most frequently persistent symptoms (brain fog characterised by difficulties in cognitive function, attention, and memory, persistent fatigue, and post‐exertion malaise).^11^

Despite these limitations, our survey provided a platform for trans people, often marginalised and underrepresented in research, to share their experiences during the COVID‐19 pandemic. As the only Australian study in this area, it provides insights into their experience of health care, including discrimination.

### Conclusion

Relatively high COVID‐19 vaccination rates among the respondents to our survey of trans people in Australia may reflect the success of targeted LGBTIQA+ health promotion and vaccination programs during the pandemic. They may also indicate that safe, trans‐affirming health care will be used by trans people if it is provided. A targeted public health response, co‐created with the trans community, could reduce the discrimination in health care and health inequity experienced by trans people, ensuring that all health professionals can provide culturally safe and affirming health care.

## Data sharing statement

De‐identified participant data are available upon reasonable request to the corresponding author (adac@unimelb.edu.au), provided that the aim of the request is deemed to be of benefit to the trans and gender‐diverse community and has received Austin Health Human Research Ethics Committee approval (as an amendment).

## Open access

Open access publishing facilitated by The University of Melbourne, as part of the Wiley ‐ The University of Melbourne agreement via the Council of Australian University Librarians.

## Competing interests

No relevant disclosures.

## Supporting information


Supplementary methods

## References

[1] Zwickl S , Wong AFQ , Bretherton I , et al. Health needs of trans and gender diverse adults in Australia: a qualitative analysis of a national community survey. Int J Environ Res Public Health 2019; 16: 5088. 31847083 10.3390/ijerph16245088 PMC6950552

[2] Bretherton I , Thrower E , Zwickl S , et al. The health and well‐being of transgender Australians: a national community survey. LGBT Health 2021; 8: 42‐49. 33297824 10.1089/lgbt.2020.0178 PMC7826417

[3] Zwickl S , Wong AFQ , Dowers E , et al. Factors associated with suicide attempts among Australian transgender adults. BMC Psychiatry 2021; 21: 81. 33557793 10.1186/s12888-021-03084-7 PMC7869522

[4] Boyer TL , Youk AO , Blosnich JR , et al. Suicide, homicide, and all‐cause mortality among transgender and cisgender patients in the veterans health administration. LGBT Health 2021; 8: 173‐180. 33544021 10.1089/lgbt.2020.0235

[5] Hughes LD , King WM , Gamarel KE , et al. Differences in all‐cause mortality among transgender and non‐transgender people enrolled in private insurance. Demography 2022; 59: 1023‐1043. 35548863 10.1215/00703370-9942002 PMC9195044

[6] Zwickl S , Angus LM , Wong AFQ , et al. The impact of the first three months of the COVID‐19 pandemic on the Australian trans community. Int J Transgend Health 2021; 24: 281‐291. 37519916 10.1080/26895269.2021.1890659 PMC10373614

[7] World Health Organization . Coronavirus disease (COVID‐19) situation report 162. 30 June 2020. https://www.who.int/docs/default‐source/coronaviruse/20200630‐covid‐19‐sitrep‐162.pdf?sfvrsn=e00a5466_2 (viewed June 2021).

[8] Zwickl S , Wong AFQ , Ginger A , et al. Trans in the pandemic: stories of struggle and resilience in the Australian trans community. Melbourne: Trans Health Research Group, University of Melbourne (Austin Health), 2022. https://www.transresearch.org.au/_files/ugd/53102e_556c291b919944f282cbd05fe5b60a4b.pdf (viewed Nov 2023).

[9] Balaji JN , Prakash S , Joshi A , Surapaneni KM . A scoping review on COVID‐19 vaccine hesitancy among the lesbian, gay, bisexual, transgender, queer, intersex and asexual (LGBTQIA+) community and factors fostering its refusal. Healthcare (Basel) 2023; 11: 245. 36673613 10.3390/healthcare11020245 PMC9859126

[10] Rich AJ , Scheim AI , Koehoorn M , Poteat T . Non‐HIV chronic disease burden among transgender populations globally: a systematic review and narrative synthesis. Prev Med Rep 2020; 20: 101259. 33335828 10.1016/j.pmedr.2020.101259 PMC7732872

[11] Australian Institute of Health and Welfare . Long COVID in Australia: a review of the literature (Cat. no. PHE 318). Canberra: AIHW, 2022. https://www.aihw.gov.au/getmedia/9592f439‐9b96‐4589‐a55d‐6b04e262e5e1/aihw‐phe‐318.pdf.aspx?inline=true (viewed Mar 2023).

[12] Wang Q , Xu R , Volkow ND . Increased risk of COVID‐19 infection and mortality in people with mental disorders: analysis from electronic health records in the United States. World Psychiatry 2021; 20: 124‐130. 33026219 10.1002/wps.20806 PMC7675495

[13] Australian Bureau of Statistics . Patient experiences. Reference period 2021–22 financial year. 18 Nov 2022. https://www.abs.gov.au/statistics/health/health‐services/patient‐experiences/latest‐release (viewed Mar 2023).

[14] Operation COVID Shield (Australian Government) . COVID‐19 vaccine roll‐out. 31 May 2022. https://www.health.gov.au/sites/default/files/documents/2022/05/covid‐19‐vaccine‐rollout‐update‐31‐may‐2022.pdf (viewed Mar 2023).

[15] Australian COVID‐19 Serosurveillance Network . Seroprevalence of SARS‐CoV‐2‐specific antibodies among Australian blood donors: round 2 update. 26 July 2022. https://www.kirby.unsw.edu.au/sites/default/files/documents/COVID19‐Blood‐Donor‐Report‐Round2‐May‐Jun‐2022%5B1%5D.pdf (viewed Mar 2023).

[16] Hembree WC , Cohen‐Kettenis PT , Gooren L , et al. Endocrine treatment of gender‐dysphoric/gender‐incongruent persons: an Endocrine Society clinical practice guideline. J Clin Endocrinol Metab 2017; 102: 3869‐3903. 28945902 10.1210/jc.2017-01658

[17] Olson‐Kennedy J , Warus J . The impact of male chest reconstruction on chest dysphoria in transmasculine adolescents and young men: a preliminary study. J Adolesc Health 2017; 60 (Suppl 1): S88.

[18] Foster Skewis L , Bretherton I , Leemaqz SY , et al. Short‐term effects of gender‐affirming hormone therapy on dysphoria and quality of life in transgender individuals: a prospective controlled study. Front Endocrinol (Lausanne) 2021; 12: 717766. 34394009 10.3389/fendo.2021.717766 PMC8358932

[19] White Hughto JM , Reisner SL . A systematic review of the effects of hormone therapy on psychological functioning and quality of life in transgender individuals. Transgend Health 2016; 1: 21‐31. 27595141 10.1089/trgh.2015.0008 PMC5010234

[20] Snoswell CL , Arnautovska U , Haydon HM , et al. Increase in telemental health services on the Medicare Benefits Schedule after the start of the coronavirus pandemic: data from 2019 to 2021. Aust Health Rev 2022; 46: 544‐549. 35732310 10.1071/AH22078

[21] Isautier JM , Copp T , Ayre J , et al. People's experiences and satisfaction with telehealth during the COVID‐19 pandemic in Australia: cross‐sectional survey study. J Med Internet Res 2020; 22: e24531. 33156806 10.2196/24531 PMC7732356

[22] Bouman WP , Schwend AS , Motmans J , et al. Language and trans health. Int J Transgend 2017; 18: 1‐6.

[23] Philip BV . Impact of Covid‐19 on transgender persons: the need for an inclusive approach. Int J Sex Health 2021; 33: 248‐267. 10.1080/19317611.2021.1906375 PMC10903614 38595748

[24] Gil RM , Freeman TL , Mathew T , et al. Lesbian, gay, bisexual, transgender, and queer (LGBTQ+) communities and the coronavirus disease 2019 pandemic: a call to break the cycle of structural barriers. J Infect Dis 2021; 224: 1810‐1820. 34323998 10.1093/infdis/jiab392 PMC9103180

[25] Thomas S. Fab jab: LGBT community steps up to get vaccinated at Pride Centre. Star Observer (Sydney), 21 Oct 2021. https://www.starobserver.com.au/news/fab‐jab‐lgbt‐community‐steps‐up‐to‐get‐vaccinated‐at‐pride‐centre/206495 (viewed Apr 2023).

[26] TransHub (ACON) . Vaccinations. Updated 7 Mar 2023. (https://www.transhub.org.au/covid/vaccination) (viewed Apr 2023).

