# Supplementary material for: Disruption of gender‐affirming health care, and COVID‐19 illness, testing, and vaccination among trans Australians during the pandemic: a cross‐sectional survey
Source: Med J Aust. 2023 Nov 23;220(1):23–8. doi: 10.5694/mja2.52169 (PMC10952718; doi:10.5694/mja2.52169)
Supplement: Supplementary file 1 — Supplementary methods [file MJA2-220-23-s001.pdf]

MJA
The Medical Journal of Australia

Supporting Information

Supplementary results

This appendix was part of the submitted manuscript and has been peer reviewed.
It is posted as supplied by the authors.

Appendix to: Zwickl S, Ruggles T, Wong AFQ, et al. Disruption of gender-affirming health care, and COVID-19 illness, testing, and vaccination among trans Australians during the pandemic: a cross-sectional survey. *Med J Aust* 2024; doi: 10.5694/mja2.52169.

Survey: Two years into the COVID-19 pandemic

LIVING SITUATION
This first set of questions asks about your living situation. If answering these questions makes you feel unsafe or distressed or you don't want to answer them, please feel free to skip them.

What postcode have you been living in for the majority of the time since July 2020?

Does anyone in your household make you feel unsafe or afraid?
- Yes
- No
- Unsure
- Prefer not to say

    If yes to unsafe, is your experience of feeling unsafe or afraid in your household related to your gender?
    - Yes
    - No
    - Unsure
    - Prefer not to say

    If yes to unsafe, Have you sought any support in regard to your safety at home? (select all that apply)
    - I have not sought any support
    - I contacted a mainstream hotline e.g., 1800 RESPECT
    - I contacted a LGBTIQA+ specific hotline
    - I spoke to a mental health professional (e.g., psychologist)
    - The police became involved
    - I have talked to a friend/family member I trust
    - I discussed it in an online group or forum
    - Other
    - Unsure
    - Prefer not to say

        You selected 'other'. Please elaborate: [free-text]

        You selected that you have not sought any support related to feeling unsafe or afraid in your household. Why have you not sought support? (select all that apply)
        - I am concerned it might escalate the situation
        - I do not have sufficient privacy (e.g., to make a phone call or have a telehealth appointment)
        - I wasn't sure if it counted as abuse/didn't want to call it abuse
        - It did not feel like I needed support
        - I did not know where to go for support
        - I don't know of any trans-affirming supports
        - I feared discrimination as a trans person
        - Other
        - Unsure
        - Prefer not to say

            You selected 'other'. Please elaborate: [free-text]

EMPLOYMENT AND FINANCIAL SITUATION
The next set of questions asks about how the COVID-19 pandemic may have affected your employment and financial situation. If answering these questions makes you feel unsafe or distressed or you don't want to answer them, please feel free to skip them.

What best describes your current employment status? (please select all that apply)
- Employed - full time
- Employed - part time
- Employed- casual
- Unemployed
- Pension
- House duties
- Volunteer
- Retired
- Student
- My visa does not allow me to work

    In the last COVID-19 survey (May-June 2020), you indicated that you had lost your job or were working reduced hours as a result of the pandemic. What best describes your current situation?
    - I found a new job working the hours that I want
    - I found a new job but I am working less hours than I want
    - I am still in the same job and still working reduced hours
    - I am still in the same job but my hours have increased back to what I want
    - I am still unemployed but looking for work
    - I am no longer looking for or able to work (e.g., I have retired)
    - Prefer not to say

In the last 3 months, has the COVID-19 pandemic put financial strain on any of the following? (select all that apply)
- Rent/mortgage
- Utilities (e.g., electricity, gas, water, internet)
- Food/groceries
- Provision of financial support to others
- Other (e.g., healthcare, medication)

Have you accessed any of the following since the start of the pandemic in March 2020? (select all that apply)
- Financial support from the government (e.g., Jobkeeper)
- Financial support from a community organisation
- Material support from a community organisation (e.g., food)
- Early withdrawal of superannuation just to cover daily living expenses
- Taken out a personal loan just to cover daily living expenses
- Financial support from family/friends
- Material support from family/friends (e.g., food)

    Participants who indicate financial strain or access to any financial or material support except government support; Have you needed financial support due to the pandemic but not been eligible for government assistance? (e.g., you are on a temporary visa)
    - Yes
    - No
    - Unsure
    - Prefer not to say

Has the COVID-19 pandemic improved your financial situation in any of the following ways? (please select all that apply)

- Increase in government payments increased my income
- Increase in work hours increased my income
- Decrease in spending (e.g., reduced use of public transport, less eating out)

If you feel comfortable, please expand on how the COVID-19 pandemic has affected your financial situation? [free-text]

GENDER-AFFIRMING HEALTHCARE

The next set of questions asks about how the COVID-19 pandemic has affected your access to gender-affirming health care. If answering these questions makes you feel unsafe or distressed or you don't want to answer them, please feel free to skip them.

Have you experienced any disruptions to hormone therapy since onset of the pandemic in March 2020? (please select all that apply)

- I have not used hormone therapy during the pandemic
- I wanted to start hormones but this process was delayed
- I had difficulty getting prescriptions for gender-affirming hormone therapy
- I had difficulty accessing supply of gender-affirming hormone therapy
- I had difficulty getting my gender-affirming hormone therapy administered (e.g., injections)
- I have not experienced any disruptions to my hormone therapy

Since the start of the pandemic in March 2020 have you experienced discrimination related to your trans status when trying to access healthcare? (select all that apply)

- Deadnaming (e.g., using your birth name rather than chosen name)
- Misgendering (e.g., using the wrong pronouns)
- Denial of care (e.g., refusing to see you because you are trans)
- Delayed care (e.g., not taking your health complaints seriously)
- Inappropriate questions related to being trans when treating an unrelated issue
- Being laughed at or joked about by a health professional
- Having a health professional make inappropriate comments about your trans status
- Being told gender-affirming healthcare isn't a priority during the pandemic

In the last COVID-19 survey (May-June 2020), you indicated that you had not experienced any cancellation or postponement of gender-affirming surgery. Have you experienced any disruptions to gender-affirming surgery since the last survey?

- Yes, my surgery was cancelled or postponed
- No, my surgery went ahead as planned
- I did not have any surgery booked

  If yes, Have you been able to have surgery since?

  - Yes
  - No, but a surgery date has been proposed
  - No, and no surgery date has been proposed
  - Prefer not to say

In the last COVID-19 survey (May-June 2020), you indicated that your gender-affirming surgery had been postponed or cancelled. Have you been able to have surgery since?

- Yes
- No, but a surgery date has been proposed
- No, and no surgery date has been proposed
- Prefer not to say

If had surgery during pandemic, Has your aftercare post-surgery been affected by the COVID-19 pandemic? (e.g., have not been able to have an in-person follow-up appointment)

- Yes
- No
- Unsure
- Prefer not to say

Have you used telehealth (telephone or video appointments) since the start of the pandemic?

- Yes
- No
- Unsure
- Prefer not to say

  If yes, compared to face-to-face appointments, how would you rate your experience? (select all that apply)

  - It made appointments more accessible (e.g., less travel, less anxiety about leaving home)
  - I found it just as effective or more effective than face-to-face
  - It made appointments less accessible (e.g., sensory difficulties, no privacy at home)
  - I found it less effective than face-to-face
  - Unsure
  - Prefer not to say

Has the COVID-19 pandemic affected your healthcare needs in other ways? If so, how? [free-text]

EXPERIENCES WITH COVID-19

The next set of questions asks about your personal experience regarding COVID-19 testing, vaccination, diagnosis, and symptoms. If answering these questions makes you feel unsafe or distressed or you don't want to answer them, please feel free to skip them.

Have you been tested for COVID-19 at a testing facility, hospital, or other clinic?

- Yes
- No
- Unsure
- Prefer not to say

  If yes to Clinic test, Was the experience of getting tested for COVID-19 trans-affirming? (select all that apply)

  - My gender was respected
  - My expression was respected
  - My name and pronouns were respected
  - Other [free-text]

  If yes to Clinic test, Did you have any experiences that were not trans-affirming or discriminatory when getting tested for COVID-19? (select all that apply)

  - My gender was not respected
  - My expression was not respected
  - My name and pronouns were not respected
  - My test results were lost or delayed which I believe was a result of mismatch in name and/or gender marker
  - I was denied testing which I believe was due to being trans
  - Other [free-text]

Have you had COVID-19?
- Yes, I tested positive for COVID-19 once
- Yes, tested positive for COVID-19 on more than one occasion
- I think so but I wasn't tested
- No
- Unsure
- Prefer not to say

  If 'I think so but I wasn't tested', Were you not tested for reasons related to being trans? (select all that apply)
  - I was concerned/feared that my gender would not be respected
  - I was concerned/feared that my expression would not be respected
  - I was concerned/feared that my name and pronouns would not be respected
  - I was concerned/feared my test results would be lost or delayed due to a mismatch in name and/or gender marker
  - I was concerned/feared that using my legal name when registering a test would be distressing
  - I was concerned/feared that I would be denied testing due to being trans
  - Other [free-text]

  If yes or think so to COVID-19, How would you describe your COVID-19 symptoms/experience?
  - Asymptomatic
  - Mild
  - Moderate
  - Severe but not hospitalised
  - I was hospitalised but did not require admission to intensive care
  - I was hospitalised and was admitted to the intensive care unit
  - Unsure
  - Prefer not to say

  If yes or think so to COVID-19, Have you experienced any long-term (3+ months) health consequences of your COVID-19 infection? (e.g. ongoing fatigue, brain fog)
  - Yes
  - No
  - Unsure
  - Prefer not to say

Have you been vaccinated against COVID-19?
- One dose
- Two doses
- Three or more doses
- No
- Prefer not to say

  If vaccinated, Where were you vaccinated?
  - Public vaccination clinic
  - LGBTIQA+ or trans-specific vaccination clinic
  - Pharmacy
  - GP Clinic
  - Other
  - Unsure
  - Prefer not to say

If vaccinated, Was the experience of getting vaccinated trans-affirming? (select all that apply)

- My gender was respected
- My expression was respected
- My name and pronouns were respected
- My vaccination certification was correct
- Other [free-text]

If vaccinated, Did you have any experiences that were not trans-affirming or discriminatory when getting vaccinated? (select all that apply)

- My gender was not respected
- My expression was not respected
- My name and pronouns were not respected
- My vaccination certification was not correct
- I had to follow up on my vaccine certification
- Other [free-text]

If vaccinated, Have you experienced any issues with proving your vaccination status due to name or gender mismatch with legal documents?

- Yes
- No
- Unsure
- Prefer not to say

If not vaccinated, Is the reason you are not vaccinated for COVID-19 related to you being trans? (select all that apply)

- I was concerned/feared that my gender would not be respected
- I was concerned/feared that my expression would not be respected
- I was concerned/feared that my name and pronouns would not be respected
- I was concerned/feared my vaccination certification would be lost or delayed due to a mismatch in name and/or gender marker
- I was concerned/feared that using my legal name when getting vaccinated would be distressing
- I was concerned/feared that I would be denied vaccination due to being trans
- Other [free-text]

DEPRESSIVE SYMPTOMS

The following 9 questions are part of a standardised measure of depression called the PHQ-9. As this is commonly used in surveys, responses to these questions allow us to compare to other survey results from different times and populations. If answering these questions makes you feel unsafe or distressed or you don't want to answer them, please feel free to skip them.

Over the last 2 weeks, how often have you been bothered by any of the following problems? [on a scale of not at all – several days – more than half the days – nearly everyday]

- Little interest or pleasure in doing things
- Feeling down, depressed, or hopeless
- Trouble falling or staying asleep, or sleeping too much
- Feeling tired or having little energy
- Poor appetite or overeating
- Feeling bad about yourself or that you are a failure or have let yourself or your family down
- Trouble concentrating on things, such as reading the newspaper or watching television
- Moving or speaking so slowly that other people could have noticed? Or the opposite – being so fidgety or restless that you have been moving around a lot more than usual
- Thoughts that you would be better off dead or of hurting yourself in some way

ANXIETY
The following 7 questions are part of a standardised measure of anxiety called the GAD-7. As this is commonly used in surveys, responses to these questions allow us to compare to other survey results from different times and populations. If answering these questions makes you feel unsafe or distressed or you don't want to answer them, please feel free to skip them.

Over the last 2 weeks, how often have you been bothered by any of the following problems? [on a scale of not at all – several days – more than half the days – nearly everyday]

- Feeling nervous, anxious, or on edge
- Not being able to stop or control worrying
- Worrying too much about different things
- Trouble relaxing
- Being so restless that it's hard to sit still
- Becoming easily annoyed or irritable
- Feeling afraid as if something awful might happen

SUICIDALITY AND SELF-HARM
The next set of questions asks about suicide attempts and self-harm during the COVID-19 pandemic. If answering these questions makes you feel unsafe or distressed or you don't want to answer them, please feel free to skip them.

Have you attempted suicide since the start of the pandemic in March 2020?
- Yes
- No
- Unsure

Have you self-harmed since the start of the pandemic in March 2020?
- Yes
- No
- Unsure

  If yes to attempt or self-harm or thoughts of suicide or self-harm (PHQ-9 Item 9), What have you found helpful to deal with thoughts of self-harm or suicide? [free-text]

SUPPORT SEEKING AND POSITIVE COPING STRATEGIES
This set of questions will ask you about what coping strategies and support seeking you are implementing during this time. If answering these questions makes you feel unsafe or distressed or you don't want to answer them, please feel free to skip them.

Have you used any of the following services during the pandemic? (select all that apply)
- National – Qlife
- National - Discharged
- National - Lifeline
- National - Beyond Blue
- National - Kids Helpline
- National - Headspace
- ACT - AIDS Action Council
- ACT - A Gender Agenda
- ACT - Meridian
- NSW - Twenty10
- NSW - The Gender Centre
- NSW - ACON
- NT - Northern Territory AIDS and Hepatitis Council
- QLD - Diverse Voices

- QLD - Queensland Council for LGBTI Health
- QLD - Open Doors Youth Service
- QLD - Qspace
- SA - Bfriend
- SA - Rainbow Directory SA
- SA - SHINESA
- TAS - Switchboard
- TAS - Working It Out
- VIC - Switchboard
- VIC - Zoe Belle Gender Centre
- VIC - Minus18
- VIC - Thorne Harbour Health (Equinox)
- VIC - Rainbow Door
- VIC - Queerspace
- WA - Living Proud
- WA - The Freedom Centre
- WA - TransFolk of WA
- WA - Western Australian AIDS Council
- WA – Discharged

Have you accessed any other services during the pandemic? Please elaborate:

Some people have reported positive effects from changes in the way we live due to the COVID-19 pandemic. Which of the following apply to you in the last two weeks? (select all)

- I have decreased social anxiety as my lived experience is now normalised
- I have decreased risk of being misgendered outside the home
- I don't feel obliged to attend events and socialise
- I have increased connectivity with people I care about
- I am making new social support connections
- I have taken up a new hobby or interest (e.g. learning to knit)
- I have more opportunities to be creative
- Events that have moved online are more physically accessible to me
- Events that have moved online are more financially accessible to me
- Other

  You selected ‘other’. Please elaborate: [free-text]

Is there anything else you want to share about your experience one year into the pandemic? This could be experiences related to your living situation, employment, financial situation, healthcare, mental and physical health or anything else that you feel important to share?
